# Retroviral RNA Dimerization: From Structure to Functions

**DOI:** 10.3389/fmicb.2018.00527

**Published:** 2018-03-22

**Authors:** Noé Dubois, Roland Marquet, Jean-Christophe Paillart, Serena Bernacchi

**Affiliations:** Architecture et Réactivité de l’ARN, UPR 9002, IBMC, CNRS, Université de Strasbourg, Strasbourg, France

**Keywords:** retrovirus, RNA, dimerization, HIV, MuLV, structure, function

## Abstract

The genome of the retroviruses is a dimer composed by two homologous copies of genomic RNA (gRNA) molecules of positive polarity. The dimerization process allows two gRNA molecules to be non-covalently linked together through intermolecular base-pairing. This step is critical for the viral life cycle and is highly conserved among retroviruses with the exception of spumaretroviruses. Furthermore, packaging of two gRNA copies into viral particles presents an important evolutionary advantage for immune system evasion and drug resistance. Recent studies reported RNA switches models regulating not only gRNA dimerization, but also translation and packaging, and a spatio-temporal characterization of viral gRNA dimerization within cells are now at hand. This review summarizes our current understanding on the structural features of the dimerization signals for a variety of retroviruses (HIVs, MLV, RSV, BLV, MMTV, MPMV…), the mechanisms of RNA dimer formation and functional implications in the retroviral cycle.

## Introduction

**FIGURE 1:**
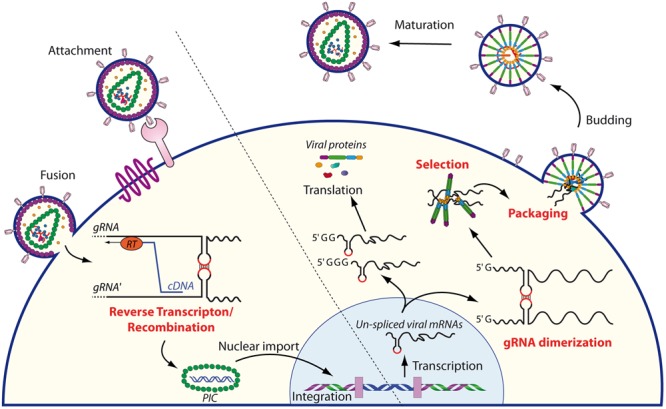
Schematic overview of the role of RNA dimerization in the retroviral life cycle. The cycle begins with the entry of the retrovirus within the target cell, followed by reverse transcription of the RNA genome into cDNA. During this step, gRNA dimerization plays an important role since RT may switch between strands, thus allowing genome repair and/or shuffling. The pre-integration complex (PIC) is then translocated into the nucleus where it is integrated in the genome of the target cell. The unspliced mRNAs are transcribed by the host machinery from the integrated provirus and transported to the cytoplasm. There, the single 5′ capped mRNAs serve as genomic RNAs that dimerize and are subsequently selected and packaged into the nascent virions, while mRNAs beginning with two or three guanosine are translated by the host machinery (Kharytonchyk et al., 2016). After budding, immature particles follow a maturation step initiated by the viral protease to produce infectious virions.

During the late phase of their replication cycle, retroviruses package two homologous copies of their genomic RNA (gRNA) in order to produce infectious viral particles. This co-packaging of gRNA molecules is highly facilitated by the dimerization signal within the 5′-region of the viral genome and by the viral Gag precursor in order to form new viral particles (Lee et al., 1999; Cimarelli et al., 2000, for reviews see D’Souza and Summers, 2005; Mailler et al., 2016). Genome dimerization is a highly conserved process amongst retroviruses, and this feature is crucial for several important steps in the retroviral life cycle (**Figure 1**). First, for several retroviruses, e.g., human immunodeficiency virus type 1 (HIV-1) and murine leukemia virus (MuLV), gRNA dimerization is critical for selective packaging of the genome (Berkhout and van Wamel, 1996; Mougel et al., 1996; Paillart et al., 1996a; McBride and Panganiban, 1997; Mougel and Barklis, 1997; Aagaard et al., 2004; Houzet et al., 2007). Second, the conformational changes induced by RNA dimerization may also regulate translation of the unspliced gRNA (Kharytonchyk et al., 2016; Boeras et al., 2017). Third, dimerization of the viral genome also plays an important role during the reverse transcription step, allowing genome repair by strand transfer when one of the two RNA strands is damaged (Mikkelsen and Pedersen, 2000). Finally, genome dimerization presents the great advantage of increasing genetic diversity by allowing genetic recombination during reverse transcription (Mikkelsen et al., 2000; Moore et al., 2009).

The first evidence for the existence of a dimeric genome came from ultracentrifugation sedimentation analysis of gRNA extracted Rous sarcoma virus (RSV) (Duesberg, 1968; Mangel et al., 1974), even though a tetrameric organization of the genome had also been proposed (Montagnier et al., 1969). Subsequent sedimentation and electron microscopy analyses supported the dimeric organization of the genome and extended this observation to other retroviruses families such as alpharetroviruses, gammaretroviruses, and lentiviruses (Kung et al., 1976; Bender et al., 1978; Maisel et al., 1978; Murti et al., 1981; Höglund et al., 1997), thus revealing the conservation and the importance of gRNA dimerization in the life cycle of retroviruses. Further studies showed that regions involved in gRNA dimerization, historically referred to as the dimer linkage structure (DLS), are typically close to the 5′-end of gRNA, and highly structured with multiple stem-loop motifs (Prats et al., 1990; Roy et al., 1990; Tounekti et al., 1992; Baudin et al., 1993; Mougel et al., 1993; Garzino-Demo et al., 1995; Jossinet et al., 2001; Abbink and Berkhout, 2003; D’Souza et al., 2004; Aktar et al., 2013, 2014). Even though retroviral genomes are rather large (from ∼7 to ∼12 kb), gRNA dimerization was observed to be mediated by relatively short sequences ranging from 50- to few hundreds of nucleotides (Kung et al., 1975; Bender et al., 1978; Maisel et al., 1978; Murti et al., 1981). *In vitro* analysis showed that DLS-containing RNA fragments could dimerize at temperatures ranging from 37 to 60°C depending on the virus, in the presence of monovalent (Na^+^ or K^+^ ranging from 0.1 to 0.3 M), and/or divalent (Mg^2+^ ranging from 1 to 10 mM) cations (Bieth et al., 1990; Darlix et al., 1990; Prats et al., 1990; Roy et al., 1990; Marquet et al., 1991; Paoletti et al., 1993). However, a better understanding of the precise mechanisms governing the dimerization process was only obtained with the identification of the HIV-1 dimerization initiation site (DIS).

A common feature of retroviral DIS is the presence of at least one short palindromic sequence enabling intermolecular base-pairing, thus forming kissing-loop structures (Laughrea and Jetté, 1994; Paillart et al., 1994; Skripkin et al., 1994; Girard et al., 1995; Muriaux et al., 1995). In the case of HIV-1, chemical modification interference assays allowed the identification of the six nucleotides constituting the DIS (Skripkin et al., 1994). Kissing-loop complexes, often referred to as “loose dimers,’ are characterized by low thermal stability (Fu and Rein, 1993; Fu et al., 1994; Jalalirad and Laughrea, 2010), and can only be visualized by gel electrophoresis under native conditions, since even mild denaturing conditions were found to dissociate RNA dimers during migration (Marquet et al., 1994; Laughrea and Jetté, 1996; Polge et al., 2000). However, incubation of DLS-containing RNAs at non-physiological high temperatures (50–60°C) was found to induce formation of RNA dimers resistant to mild-denaturing electrophoresis conditions and were thus called “tight dimers.” Importantly, tight dimers are also obtained at physiological temperature in the presence of the cognate nucleocapsid (NC) protein (Girard et al., 1996; Muriaux et al., 1996), well-known for its RNA chaperone properties (Feng et al., 1996; Girard et al., 1996; Muriaux et al., 1996; Rist and Marino, 2002; Mujeeb et al., 2007; Aduri et al., 2013; for a review Levin et al., 2005). These results lead to the notion that NC lowers the energy barriers and promote refolding of the 5′-end region of gRNA into a more stable conformation (Feng et al., 1996; Gorelick et al., 1996, 1999; Cruceanu et al., 2006). Studies on short sequences harboring the DIS suggested that these conformational changes could involve the refolding of these structures by forming cruciform intermediates that evolve into extended intermolecular base-pairing (Polge et al., 2000; Rist and Marino, 2002; Bernacchi et al., 2005).

*In vivo*, subsequent to viral budding, immature virions undergo a maturation step that is mandatory for viral infectivity and is mediated by the viral protease that sequentially cleaves the viral Pr55^Gag^ and Pr160^GagPol^ precursors into the mature structural and enzymatic proteins. Concomitantly to this proteolytic maturation, the viral genome undergoes a maturation process (Ohishi et al., 2011; Mailler et al., 2016). Indeed, HIV-1 and MuLV gRNA dimers extracted from immature viral particles are less stable than those extracted from mature virions (Fu and Rein, 1993; Fu et al., 1994; Jalalirad and Laughrea, 2010; Ohishi et al., 2011; Grohman et al., 2014). Interestingly, the different stabilities observed in immature and mature virions are very similar to those observed *in vitro* for loose and tight RNA dimers, respectively, suggesting these conformations may reflect the maturation process of gRNA into viral particles.

In this review, we will focus on our current understanding of the mechanisms and molecular factors involved in gRNA dimerization for different retrovirus families both *in vitro* and *in cellula*, its role during the retroviral life cycle, and finally its potential targeting by molecules aimed at inhibiting viral replication.

## Alpha-Retroviruses Genome Dimerization

**FIGURE 2:**
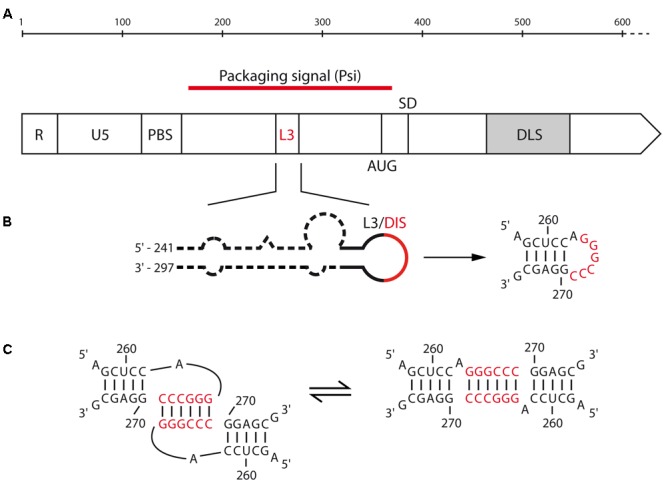
Motifs involved in the RNA dimerization of alpha-retrovirus. **(A)** Schematic representation of the 5′-end of alpha retrovirus gRNA. The functional domains and their positions are represented: R, repeated region; U5, unique sequence in 5′; PBS, primer binding site; AUG, *gag* translation initiation codon; SD, splice donor site; L3 and DLS, dimerization motifs for ALV and RSV, respectively. **(B)** Predicted secondary structure of the ALV L3 stem-loop. The consensus nucleotides are represented, with the palindromic hexanucleotide sequence highlighted in red. **(C)** Proposed kissing-loop complex and extended duplex conformations of ALV L3 element.

Even though the first evidence for retroviral RNA dimerization came from sedimentation and electron microscopy studies of alpha-retroviruses such as RSV (Duesberg, 1968; Canaani et al., 1973; Mangel et al., 1974), the precise mechanisms underlying this process remains surprisingly poorly defined in comparison with other model retroviruses such as HIV-1 and MuLV. Strikingly, while most DLS/DIS are found within the 5′-untranslated region (UTR) of gRNA, electron micrographs of gRNA dimers extracted from RSV virions located the DLS within the *gag* gene, around position 480–540 from the 5′ end (**Figure 2A**) (Murti et al., 1981). One other peculiarity lies in the fact that RSV DLS contains an imperfect palindrome that was first proposed to contribute to RNA dimerization (Schwartz et al., 1983). In rather good agreement with these findings, *in vitro* analyses of the dimerization of the first 634 nucleotides (nts) of RSV gRNA suggested that the DLS would be located between positions 544–564 (Bieth et al., 1990; Lear et al., 1995) and would likely involve Watson–Crick base-pairing of the palindrome mentioned above (Lear et al., 1995). However, analysis of gRNA dimerization of the avian sarcoma-leucosis virus (ASLV), another alpha-retrovirus, relies on the L3 element, a conserved 19-nts hairpin harboring a perfect palindrome sequence in its apical loop and located upstream of the major Splice Donor (SD) site (**Figure 2B**) (Fossé et al., 1996). Interestingly, in the absence of NC, viral RNA fragments of 626 nts in length form loose dimers at 37°C, involving loop-loop interactions through the L3 element (**Figure 2C**, left) (Polge et al., 2000). However, when incubated at 60°C, these fragments formed tight dimers, and heterodimerization of L3 stem mutants supported the notion that *in vitro* ASLV gRNA tight dimers are characterized by the formation of an extended duplex (**Figure 2C**, right) (Polge et al., 2000).

## Beta-Retroviruses Genome Dimerization

**FIGURE 3:**
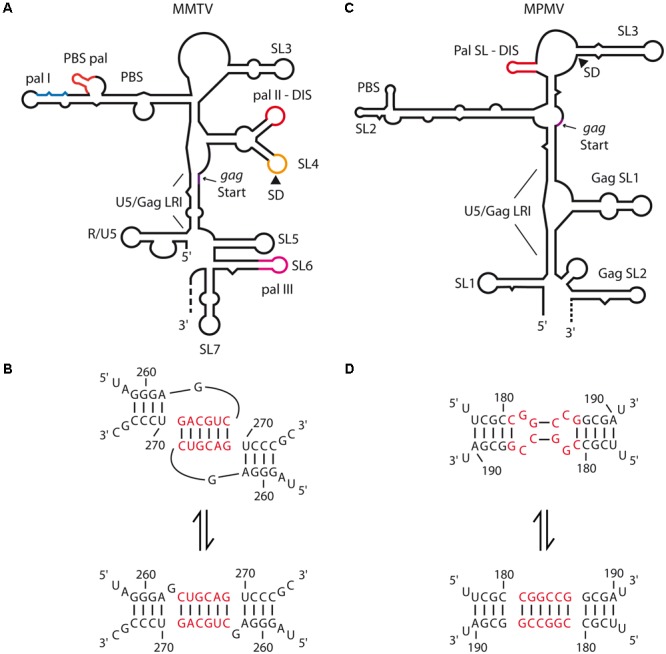
Secondary structure of the 5′-end of MMTV and MPMV genomic RNAs and dimerization models. **(A)** MMTV gRNA secondary structure, the different palindromic sequence (pal I, II, III, and PBS-pal) and the long-range interaction (LRI) between U5 and the beginning of *gag* are indicated. The different stem-loops (SL) are numbered as proposed by (Aktar et al., 2014). SL4 comprises both pal II which is the proposed DIS, and the major SD site. The R and PBS regions are also represented. **(B)** MMTV pal II switch models from kissing-loop complex to extended duplex. The nucleotide positions are represented and the hexanucleotide palindrome is highlighted in red. **(C)** MPMV gRNA secondary structure, the different stem-loops (SLs) are numbered as proposed by (Aktar et al., 2013), the LRI between U5 and the beginning of *gag* is also represented. The palindromic sequence folded in a short hairpin (Pal SL) which is the proposed DIS is highlighted in red. **(D)** MPMV Pal SL switch models from the kissing-loop complex to the extended duplex. The nucleotide positions are represented and the hexanucleotide palindromic sequence is highlighted in red.

Recently, Rizvi and collaborators provided the first *in vitro* analyses of structure and dimerization mechanisms of the 5′-end region of the mouse mammary tumor virus (MMTV) and Mason-Pfizer monkey virus (MPMV) gRNAs (Aktar et al., 2013, 2014; Kalloush et al., 2016). Selective 2′-hydroxyl acylation analyzed by primer extension (SHAPE) and *in vitro* dimerization assays of MMTV gRNA fragments revealed that loose dimer formation is potentially mediated by two palindromic sequences, respectively, within the primer binding site (PBS-Pal) and in a bifurcated stem-loop structure (SL4) located between the PBS and the translation initiation codon of *gag* (Pal II) (**Figure 3A**). However, Pal II is the main DIS since its mutation had a greater impact on RNA dimerization than mutation of the PBS-Pal (**Figure 3B**) (Aktar et al., 2014). In MPMV, the palindromic sequence that functions as the gRNA DIS folds into a short hairpin (Pal SL) (**Figures 3C,D**) (Aktar et al., 2013). These motifs are highly conserved in MMTV and MPMV strains and their mutation leads to severe gRNA packaging and viral replication defects (Jaballah et al., 2010; Aktar et al., 2014). Interestingly, secondary structures of the 5′-end of both MPMV and MMTV gRNAs present long-range interactions (LRI) involving the 5′ unique (U5) region and a region spanning the *gag* translation initiation codon (**Figures 3A,C**) (Aktar et al., 2014; Kalloush et al., 2016), similarly to what has been observed for lentiviruses, for which such interaction was proposed to promote gRNA dimerization (see below). In MPMV, these LRIs are required for gRNA packaging and viral propagation, even though mutations destabilizing LRIs have only modest effects on RNA dimerization (Kalloush et al., 2016).

## Gamma-Retroviruses Genome Dimerization

**FIGURE 4:**
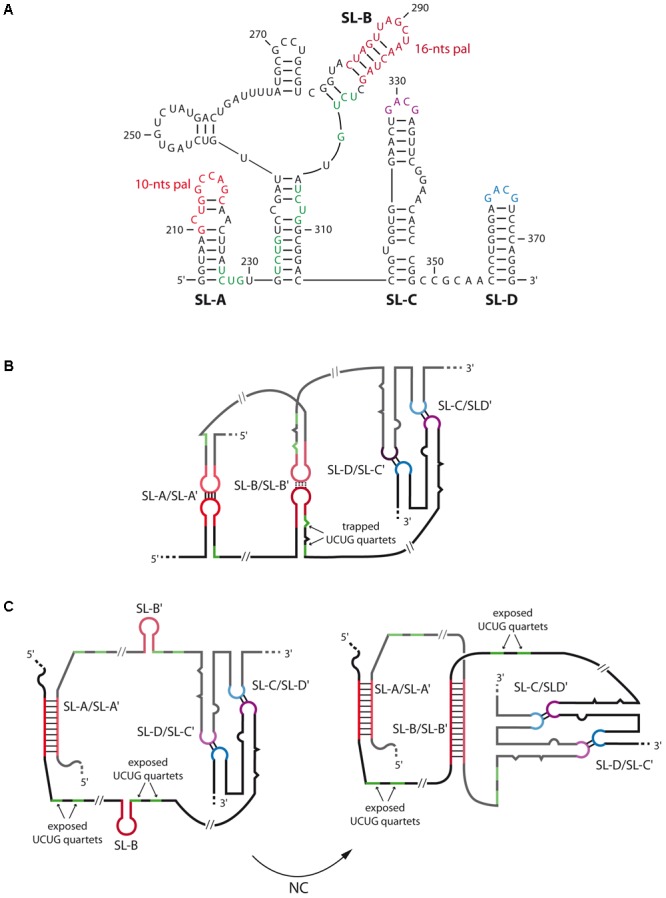
Secondary structures and dimerization of MuLV genomic RNA. **(A)** Secondary structure of the 5′-end of MuLV gRNA containing the four stem-loops SL-A to SL-D. The nucleotide positions are indicated. **(B)** *In vitro* model of the kissing-loop/loose dimer complex. SL-A and SL-B contain a palindromic sequence of 10 and 16-nts long, respectively (in red), that promote the initiation of RNA dimerization. SL-C (purple) and SL-D (blue) both present a GACG tetraloop involved in heterologous non-canonical loop-loop interactions that stabilize the duplex. In this conformation, several UCUG quartets (green), which constitute high affinity binding sites for the viral NC protein, are trapped within SL-A and SL-B. **(C)** Secondary structure model of gRNA dimer maturation steps derived from SHAPE data (Grohman et al., 2014): the extracted *ex viro* immature form (left) is converted by NC into the mature 5′-end RNA dimer (right). In the immature conformation, only SL-A and SL-A′ are paired and adopt an extended conformation while the SL-B elements are unpaired. This conformation exposes the UCUG quartets. In the mature conformation, both SL-A/SL-A′ and SL-B/SL-B′ loop–loop interactions adopt an extended duplex structure, thus increasing dimer stability (Gherghe et al., 2010). This model also exposes the UCUG quartets. The mature dimer is thought to be to be similar to *in vitro* 5′-end tight dimer structure.

A general feature of gamma-retroviruses RNA dimerization is the presence of several palindromes within stem-loops that contribute to dimer formation at different degrees. Within this family, murine leukemia virus (MuLV) is the most studied model, and its minimal DLS corresponds to a 170-nts long region containing four stem-loops (SL-A to SL-D) each contributing to the dimerization process (Prats et al., 1990; Roy et al., 1990; Tounekti et al., 1992; Mougel et al., 1993; Torrent et al., 1994; Girard et al., 1995; De Tapia et al., 1998; Badorrek and Weeks, 2005) (**Figure 4A**). However, comparison of the chemical reactivity between monomer and dimer also suggested that *gag* region could contribute to dimerization in a full-length genome context (Mougel et al., 1993). The first two stem-loops, SL-A and SL-B, contain palindromes of 10- and 16-nts, respectively, both critical for the *in vitro* dimerization process (**Figures 4B,C**) (Tounekti et al., 1992; Girard et al., 1995, 1996; Oroudjev et al., 1999; Ly and Parslow, 2002). Additionally, NMR and SHAPE analyses of MuLV DLS indicated that SL-C and SL-D, both presenting a highly conserved GACG apical tetraloop (Konings et al., 1992) (**Figure 4A**), form canonical heterologous loop–loop interactions that significantly contribute to dimer stability (De Tapia et al., 1998; Kim and Tinoco, 2000; D’Souza et al., 2004; Badorrek et al., 2006; Gherghe and Weeks, 2006; Miyazaki et al., 2010b) (**Figure 4B**). While MuLV initial loose dimer involves only few intermolecular base-pairs in the apical loops of SL-A and SL-B, *in vitro* analysis indicated that, when RNA fragments containing MuLV DLS are incubated at 60°C or at 37°C in the presence of NC, both contacts undergo structural rearrangements, and expand to the whole SL-A and SL-B palindromes (**Figure 4C**, right) (Roy et al., 1990; Girard et al., 1996; Ly and Parslow, 2002; Badorrek and Weeks, 2006). Interestingly, formation of the extended duplex exposes UCUG quartets that are base-paired in the monomeric structure (compare **Figures 4A** with **4C**), and that correspond to high affinity binding sites for NC protein. Furthermore, electron tomography and hydroxyl footprinting data both indicated that MuLV DLS adopts a compact structure upon dimerization (Badorrek et al., 2006; Badorrek and Weeks, 2006; Miyazaki et al., 2010b) suggesting that the stability of tight RNA dimers is also mediated by stacked helices, in addition to formation of the extended duplex.

*In viro* SHAPE analyses on extracted gRNA from mature MuLV particles also revealed that the gRNA dimer adopts the SL-A/SL-A′ and SL-B/SL-B′ extended duplexes conformation (Gherghe et al., 2010) (**Figure 4C**, right). Interestingly, recent chemical probing data on extracted gRNA dimers from immature MuLV particles indicated that the SL-B element would not be involved in the inter-molecular duplex (**Figure 4C**, left) (Grohman et al., 2014), thus supporting the notion of a conformational switch between immature and mature RNA dimer forms. This structural switch is likely to occur through the chaperone activity of MuLV NC, and is further facilitated by the stabilization of the duplex by SL-C and SL-D, as previously suggested (D’Souza et al., 2004; Badorrek et al., 2006; Miyazaki et al., 2010b). These data are in good agreement with findings showing that deletion of SL-A significantly decreased MuLV gRNA packaging and affected replication kinetics in cell culture, while mutation/deletion of SL-B only presented moderate effect regarding these processes (Aagaard et al., 2004). Taken together, these results suggest that only the 10-nts palindromic sequence within SL-A is crucial to form the immature gRNA dimer (**Figure 4C**, left).

Finally, *in vitro* analysis of the gRNA dimer of feline endogenous RD-114, another gamma-retrovirus, revealed the presence of several palindromic stem-loops (psl-1 to psl-5) leading to RNA dimer formation (Kharytonchyk and Pedersen, 2010). Interestingly, and similarly to MuLV, the RD-114 DLS also contains two GACG tetraloops that are close to the crucial DIS elements and contribute to *in vitro* RNA dimerization (Kharytonchyk and Pedersen, 2010).

## Delta-Retroviruses Genome Dimerization

Site-directed mutagenesis, antisense oligonucleotides mapping experiments, as well as structural predictions identified the DLS of bovine leukemia virus (BLV) in the 5′-region of the genome, in a region overlapping U5, the PBS and 30 bases downstream of this latter (Katoh et al., 1993). Interestingly, the DLS of BLV also overlaps the Psi (packaging signal), similarly to other retroviruses, since deletion of this region completely abrogated genome encapsidation and viral replication (Mansky et al., 1995). However, a particular feature of BLV is the fact that *in vitro* dimerization of gRNA is promoted by the viral matrix protein, and not by NC, contrary to what is observed for other retroviruses (Katoh et al., 1991, 1993).

**FIGURE 5:**
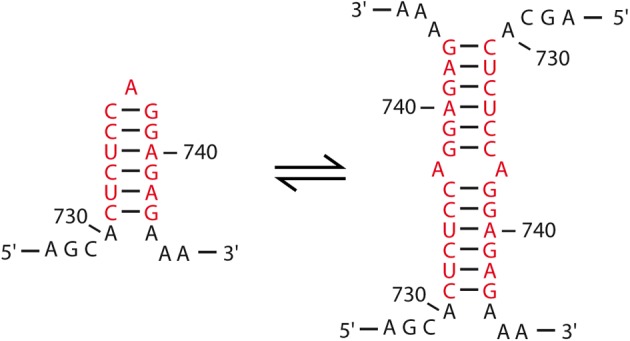
HTLV-I DIS model. Contrary to what is observed for other retroviruses, the DIS of HTLV-I presents a peculiar secondary structure harboring a single A residue in the apical loop (Monie et al., 2004). The palindrome is highlighted in red and the nucleotide positions are indicated. The proposed dimer interaction is also represented (Monie et al., 2001).

Similar experimental approaches revealed that a 32-nts sequence just upstream the PBS mediates *in vitro* dimerization of human T-cell leukemia virus type-1 (HTLV-1) gRNA (Greatorex et al., 1996). Within this region, a conserved 14-nts palindromic stem-loop sequence was identified as the DIS (Greatorex et al., 1996; Monie et al., 2001), since deletion of this motif abrogated *in vitro* RNA dimerization. Since HTLV-1 RNA dimers present a very high thermal stability (Tm ∼ 70–80°C), it was proposed that Watson–Crick base-pairings could not be solely responsible for RNA dimerization, and that other non-canonical or tertiary interactions would further contribute to the *in vitro* RNA dimer stability (Greatorex et al., 1996). Interestingly, biochemical structural analysis of this motif also indicated that, unlike other retroviruses displaying DIS larger loops, the HTLV-1 DIS forms a hairpin with a single A residue in the apical loop closed by non-canonical C-synG base-pairs (**Figure 5**) (Monie et al., 2004). However, in contrast to other retroviruses, deletion of the 37-nts DLS only caused a modest decrease in viral infectivity (20–25%) (Le Blanc et al., 2000). Therefore, further studies will be necessary to identify all the determinants involved in these processes.

## Lentiviruses Genome Dimerization

**FIGURE 6:**
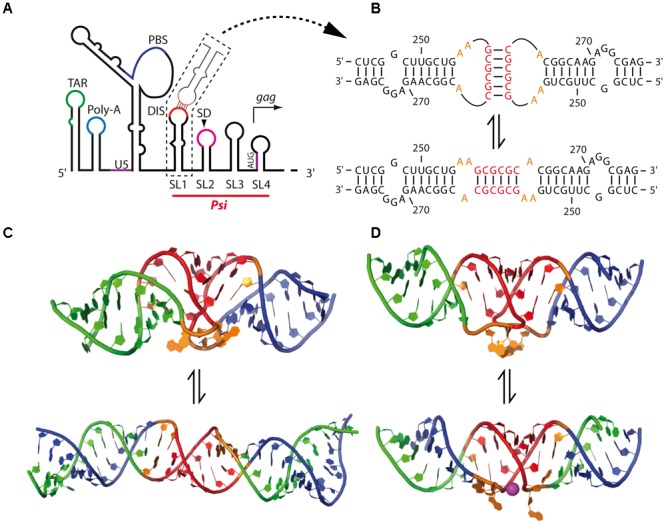
Mechanism of HIV-1 RNA dimerization. **(A)** Schematic secondary structure model of the 5′-end region of the HIV-1 gRNA. TAR, *trans*-activation response element; Poly-A, stem-loop containing the 5′-copy of the polyadenylation signal in the apical loop; U5, unique in 5′; PBS, primer binding site; DIS, dimerization initiation site; SL1–4: stem-loops 1–4 containing the Dimerization Initiation Site (DIS), the major Splice Donor (SD) site, the historical packaging signal, and the *gag* AUG initiation codon, respectively. The packaging signal (Psi) region, spanning SL1-SL4 elements, is indicated. **(B)** Model of the SL1 switch from the kissing-loop complex to the extended duplex conformation. The nucleotide positions are indicated. **(C)** Solution structures of SL1 23-mer kissing-loop complex (KC) (*up*) (Kieken et al., 2006) and of SL1 35-mer extended duplex (ED) (*down*) (Ulyanov et al., 2006) as determined by NMR. The DIS palindromic sequences are highlighted in red, and the purines flanking the DIS are in orange. Structures were drawn using the coordinates deposited on the PDB (PDB ID: 2F4X – KC – and 2GM0 – ED). **(D)** X-ray crystal structures of SL1 23-mer both in KC (*up*) (Ennifar et al., 2001) and ED (*down*) (Ennifar et al., 1999) conformations. The DIS palindromic sequences are highlighted in red. One Mg^2+^ ion found in the ED crystal structure exposing the highly conserved purines flanking the DIS (in orange) is drawn in purple. Structures were drawn using the coordinates deposited on the PDB (PDB ID: 2B8R – KC – and 2F4X – ED).

The dimerization of lentiviral RNA genomes is the best characterized retroviral dimerization process, and several important features for gRNA dimerization are conserved in the genus. Like in all retroviruses, the DLS is located at the 5′ region of the viral genome. The DIS of primate lentiviruses such as HIV-1/2 or simian immunodeficiency virus (SIV) is located within the 5′-UTR, downstream of the PBS (Skripkin et al., 1994; Dirac et al., 2001; Whitney and Wainberg, 2006) (**Figure 6A**). Contrary to gamma-retroviruses, the lentiviral DIS consists in a single palindromic sequence, typically located within the apical loop of a hairpin structure and forming homologous intermolecular canonical Watson–Crick base-pairs (**Figure 6B**). This initial intermolecular contact generally expands to a larger sequence during the stabilization of the tight dimer (**Figure 6**). Interestingly, the DIS of feline immunodeficiency virus (FIV) seems to be located within the *gag* coding region (Kenyon et al., 2011), although a structural study proposed an alternative hairpin harboring a less conserved palindromic sequence upstream of *gag* as a potential DIS (James and Sargueil, 2008).

**FIGURE 7:**
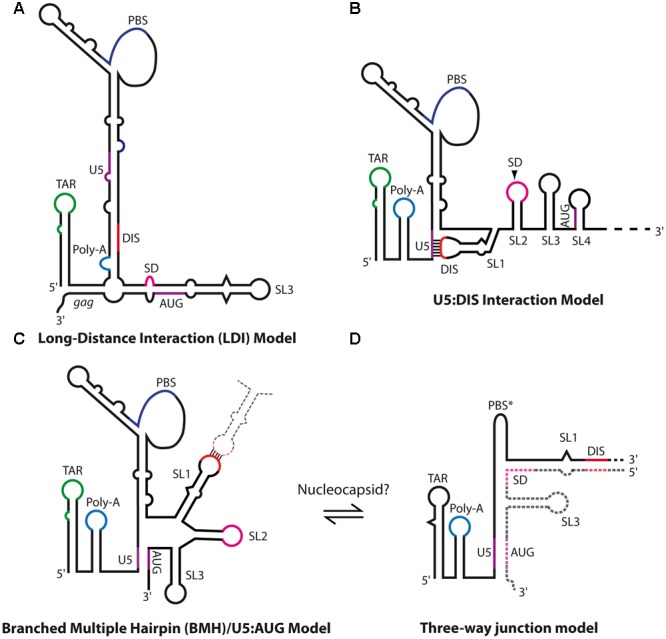
Secondary structure models of HIV-1 genomic RNA. **(A)** HIV-1 long-distance interaction model (LDI) as proposed by Huthoff and Berkhout (2001), in which SL1 DIS is base-paired together with the poly-A element. **(B)** HIV-1 U5:DIS model proposed by Lu et al. (2011), in which SL1 DIS is base-paired with the U5 region. This model was proposed to promote the translation of unspliced gRNAs by repressing dimerization (Lu et al., 2011). **(C,D)** Dimerization-competent structural models of HIV-1 5′-end. **(C)** The branched multiple hairpin (BMH)/U5:AUG models (Huthoff and Berkhout, 2001; Lu et al., 2011) in which SL1 DIS is exposed while U5 base-pairs with the region overlapping *gag* translation initiation codon, which promotes dimerization and was proposed to repress translation (Lu et al., 2011). **(D)** Keane et al. (2015) recently proposed a putative three-way junction structure of the extended duplex conformation in which the whole region downstream of SL1 is exchanged. This conformation was proposed to be achieved through NC chaperone activity.

**FIGURE 8:**
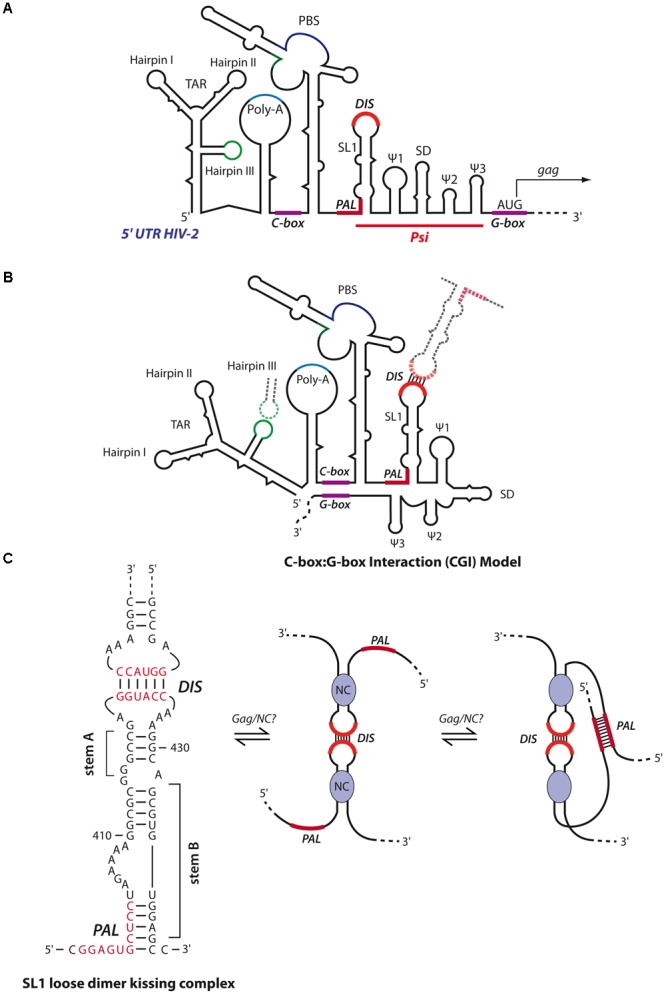
Secondary structure models of HIV-2 genomic RNA. **(A)** Schematic representation of the secondary structure model of the 5′- region of the HIV-2 gRNA. The red line delimits the packaging signal (Psi) region containing the five stem-loops SL1, Ψ1 to Ψ3 and SD. Similar to HIV-1, SL1 contains in its apical loop a hexanucleotide palindromic sequence constituting the DIS. SL1 also contains in its basal part a 10-nts palindromic sequence partially entrapped (PAL). The *gag* translation initiation codon is located within a G-rich region (G-box). Upstream of Psi, are found the TAR, the poly-A, a C-rich region important for RNA dimerization (C-box) and the PBS. **(B)** In the CGI dimer structural conformation, the C-box and G-Box are base-paired. This conformation restricts HIV-2 RNA dimerization and is adopted by loose dimers when the DIS and TAR hairpin III are involved in kissing-loop interactions (Lanchy et al., 2003a,b). **(C)** The RNA switch model from loose to tight dimer, as proposed by Purzycka et al. (2011). HIV-2 SL1 stem B is melted through Gag/NC chaperone activity, freeing the PAL region that can thus forms additional intermolecular base-pairings to stabilize the RNA dimer.

Another conserved feature in lentiviruses is the existence of a conformational switch regulating gRNA dimerization. Indeed, gRNA fragments encompassing the 5′ region of the genome adopt alternative structures involving long-range interactions (LRI) between the R/U5 elements and regions overlapping the *gag* translation initiation codon (see below for HIV-1) to expose the DIS and promote RNA dimerization (**Figure 7**), or alternatively to prevent it (**Figure 8**) (Huthoff and Berkhout, 2001; Abbink and Berkhout, 2003; Lanchy et al., 2003a,b; Whitney and Wainberg, 2006; Kenyon et al., 2008, 2011; Lu et al., 2011; Tran et al., 2015).

### Human Immunodeficiency Virus Type 1 gRNA Dimerization

The HIV-1 DIS is located in the 5′ region of the gRNA, within the packaging signal, Psi, which is composed of four stem-loops (SL1–SL4) (**Figure 6A**) (Lever et al., 1989; Aldovini and Young, 1990; Clavel and Orenstein, 1990; Clever et al., 1995; Mailler et al., 2016), although SL4, encompassing *gag* initiation codon, seems to be poorly stable and would be most likely in equilibrium with the U5 region as discussed below (Abbink and Berkhout, 2003; Lu et al., 2011). Importantly, SL1 contains in its apical loop a 6-nts palindromic sequence that have been shown by interference of chemical modifications and site-directed mutagenesis analyses to be responsible for the *in vitro* gRNA dimerization through formation of a loop-loop kissing complex (**Figure 6B**, up) SL4 (Marquet et al., 1994; Paillart et al., 1994; Skripkin et al., 1994) and is important for viral replication (Berkhout and van Wamel, 1996; Paillart et al., 1996a; Clever and Parslow, 1997; Laughrea et al., 1997, 1999). The DIS of HIV-1 is submitted to strong selection pressure, and its diversity is rather limited *in vivo*, with a strong prevalence for three GC-rich palindromes (GCGCGC, GUGCAC, and GUGCGC) (Skripkin et al., 1994; Berkhout, 1996; Paillart et al., 1996b; Hussein et al., 2010). Accordingly, *in vitro* selection experiments from randomized DIS mutants indicated that selected sequences contain the central two G-C base-pairs that are critical for kissing complex stability, while mutants presenting more than one A-U base-pair were poorly replicating (Clever et al., 1996; Laughrea et al., 1999; Lodmell et al., 2000, 2001; Hussein et al., 2010).

In HIV-1, the conformational switch from the kissing-loop complex to the extended duplex was extensively studied *in vitro* using 23-mer (corresponding to SL1 upper part) and 35-mer (corresponding to the whole SL1 hairpin) SL1 RNA fragments (**Figure 6B**). Both 3D structures were solved by NMR (Kieken et al., 2006; Ulyanov et al., 2006) (**Figure 6C**) and X-ray crystallography (Ennifar et al., 1999, 2001) (**Figure 6D**). Crystal structures of SL1 dimers revealed that Mg^2+^ ions promote exposure of two conserved unpaired purine residues flanking the DIS (Ennifar et al., 1999) (**Figure 6D**) that play an important role in the rate of NC-catalyzed duplex formation and were proposed to be involved in non-canonical intermolecular interactions (Paillart et al., 1997; Mihailescu and Marino, 2004; Mundigala et al., 2014). Interestingly, only the HIV-1 DIS containing the flanking purines can replace the cognate beta-retroviruses palindromic sequence (Aktar et al., 2013, 2014), further supporting the importance of these purine residues in HIV-1 palindrome dimerization, even in a heterologous context.

Although it has been proposed, based on the crystal structure of the SL1 23-mer dimer (**Figure 6D**), that the extended duplex formation may be the result of an intermolecular *trans*-esterification reaction (Ennifar et al., 2001), NMR and UV-melting analyses on SL1 35-mers indicated that the transition from loose to tight dimers involves melting of the SL1 upper stem, without melting the kissing-loop duplex interface, followed by interstrand exchange as refolding of the stem occurs (Mundigala et al., 2014). This model is supported by several studies showing that the SL1 internal loop destabilizes the SL1 upper stem (Takahashi et al., 2000; Greatorex et al., 2002; Baig et al., 2007; Mujeeb et al., 2007) and constitutes a NC binding site that was shown to promote NC-mediated transition from the kissing-loop complex to the extended duplex (Rist and Marino, 2002; Hagan and Fabris, 2007). Interestingly, the SL1 internal loop was recently found to constitute a major binding site for Pr55^Gag^ (Abd El-Wahab et al., 2014; Smyth et al., 2015; Bernacchi et al., 2017), suggesting that formation of the extended duplex could be promoted by the NC domain within Pr55^Gag^ as well. Moreover, Mg^2+^ ions strongly impact the rate at which the duplex is formed (Bernacchi et al., 2005) and were observed to bind the SL1 internal loop, possibly preventing premature transition from the kissing-loop to the extended duplex (Sun et al., 2007). Therefore, one could speculate on a model where Mg^2+^ and Pr55^Gag^ (or NC) compete for binding to the SL1 internal loop, thus stabilizing or destabilizing the upper stem. Consistent with this notion, the SL1 internal loop was shown to be important for RNA dimerization, packaging and viral infectivity in cell cultures (Clever et al., 1996; Paillart et al., 1996a; Clever and Parslow, 1997; Harrison et al., 1998; Laughrea et al., 1999; Shen et al., 2000) and stabilization of the SL1 hairpin resulted in replication defects (van Bel et al., 2014).

The existence of an extended duplex in large HIV-1 RNA fragments encompassing the whole DLS constitutes a question of debate and remains to be clearly demonstrated in the viral context. First, probing techniques cannot discriminate intramolecular from intermolecular base-pairings, thus making difficult the discrimination of both conformations. Second, SL1 *trans*-complementary mutants that are not able to form the extended duplex efficiently dimerized *in vitro* and were found to be as stable as wild-type RNA fragments (Paillart et al., 1996c). Besides, the conformational switch from the kissing-loop complex to the extended duplex in full-length gRNA or even in complete 5′-UTR context presents huge topological and steric constraints. Therefore, it is reasonable to consider that HIV-1 RNA tight dimer formation would rather be the result of conformational rearrangements at a larger scale, with the formation of additional contacts (see below). The notion of additional inter-genomic contacts outside SL1/DIS is also supported by electron microscopy studies on full-length gRNA dimers extracted from virions and atomic force microscopy (AFM) analysis on gRNA fragments encompassing the first 744 nts of the HIV-1 genome showing that HIV-1 gRNA dimers are not Y-shaped, like in other retroviruses, but rather formed a loop toward the genome 5′-end (Höglund et al., 1997; Andersen et al., 2004; Pallesen, 2011). Besides the DIS, the most extensively described gRNA motif potentially having a role in the dimerization process is the TAR element at the 5′ extremity of the genome (**Figure 6A**). Although HIV-1 TAR contains a 10-nts palindromic sequence in its apical loop that mediates dimerization of a 57-nts long TAR fragment (Andersen et al., 2004), it seems this link with gRNA dimerization is rather indirect. Indeed, even though electrophoretic mobility analyses of mutant TAR RNA dimers extracted from virions presented dimerization defects (Song et al., 2008; Jalalirad et al., 2012), compensatory mutations failed to restore the putative intermolecular TAR-TAR interaction (Jalalirad et al., 2012). Additionally, other *in cellula* and *in vitro* studies indicated that TAR has a moderate effect on RNA dimerization (Das et al., 2007; Lu et al., 2011; Heng et al., 2012), suggesting that TAR destabilization impacts the overall gRNA structure, thus inducing aberrant gRNA dimerization and packaging (Ooms et al., 2004; Vrolijk et al., 2008; Das et al., 2012). Therefore, the intermolecular TAR-TAR interactions would promote gRNA structure stability rather than RNA dimerization.

HIV-1 gRNA can alternatively serve as a template for the synthesis of Gag precursors, and/or be selected for encapsidation within virions as dimers. Consistent with this notion, it was suggested that the regulation of HIV-1 gRNA translation or dimerization and packaging would be due to RNA structural switches. Using biochemical and chemical probing, Huthoff and Berkhout (2001), Berkhout et al. (2002), Abbink and Berkhout (2003), Abbink et al. (2005) proposed an RNA-switch mechanism from a long-distance interaction (LDI) to a branched multiple hairpin (BMH) conformation that would regulate gRNA dimerization and translation (**Figures 7A,B**). In the LDI conformation, SL1 is disrupted and base-paired with the poly-A element, thus sequestrating the DIS sequence and potentially promoting translation (**Figure 7A**). Alternatively, in the BMH conformation, the *gag* AUG initiation codon together with the U5 region forms the so-called U5:AUG interaction, displaying SL1-SL3 to fold hairpin structures (**Figure 7B**) and promoting gRNA dimerization. Importantly, if the U5:AUG interaction is supported by phylogenic studies showing its conservation and co-variation throughout HIV-1 isolates (Abbink and Berkhout, 2003; Russell et al., 2003; Damgaard et al., 2004; Song et al., 2008; Wilkinson et al., 2008; Lu et al., 2011; Sakuragi et al., 2012; Keane et al., 2015, 2016; Tran et al., 2015; Mueller et al., 2016) and other related lentiviruses such as SIV (Whitney and Wainberg, 2006; Tran et al., 2015) or HIV-2 (see below), the LDI conformation has never been characterized *in vivo*, and RNA mutants aiming to disrupt LDI failed to affect HIV-1 unspliced RNA translation (Abbink et al., 2005), thus questioning the biological relevance of the LDI conformation. Also, mutational analysis of two highly conserved sequences, upstream of SL1 and downstream of SL4, suggested that these are part of a pseudoknot-like conformation contributing to HIV-1 DLS structure and promoting dimerization (Sakuragi et al., 2012).

An alternative *in vitro* RNA conformational switch model derived from the previous one was proposed by the group of Michael Summers by using a new NMR approach (long-range probing by adenosine interaction detection or lr-AID) which is based on selective isotopic labeling of each RNA molecule in the dimer thus allowing the discrimination between intra- and inter-molecular base-pairs (Lu et al., 2011). In this model, contrary to the LDI-BMH conformational switch, the DIS and *gag* initiation codon are alternatively base-paired with the U5 region (**Figures 7B,C**), promoting translation or RNA dimerization, respectively. Even though the dimerization competent U5:AUG RNA structure appears rather similar to the BMH conformation (Lu et al., 2011) (**Figure 7B**), a striking difference corresponds to the three-way junction RNA structure at the base of SL1, in which SL2 base-pairs with the bottom region of the PBS from the other gRNA molecule (**Figure 7D**) (Keane et al., 2015, 2016). However, this latter conformation was very recently challenged by a phylogenic study supporting the existence of SL2 hairpin structure (Mueller et al., 2016).

### Human Immunodeficiency Virus Type 2 gRNA Dimerization

The secondary structure and *in vitro* dimerization properties of HIV-2 gRNA present similarities to HIV-1 gRNA (**Figure 8A**). First, loose dimer formation involves several contacts including the SL1 region of Psi. HIV-2 SL1 also contains a 6-nts palindromic sequence within the apical loop driving the dimerization of 5′ RNA fragments (Dirac et al., 2001). Second, the HIV-2 TAR hairpin III (**Figures 8A,B**) palindromic sequence was also shown to contribute to the stabilization of the kissing-complex by forming homologous intermolecular base-pairs (**Figure 8B**) (Purzycka et al., 2011). Interestingly, in the context of RNA fragments encompassing the whole 5′-UTR (up to position ∼560), another palindromic sequence in the PBS was also described to be required for RNA dimerization (Jossinet et al., 2001; Lanchy and Lodmell, 2002; Lanchy et al., 2003b).

However, *in vitro* tight dimers of HIV-2 RNA fragments seem to be quite different from HIV-1. Indeed, RNA fragments encompassing the whole 5′-UTR, in the absence of HIV-2 NC protein and at physiological temperature, were shown to form only loose dimers through the PBS palindrome (Jossinet et al., 2001; Lanchy and Lodmell, 2002; Lanchy et al., 2003b), while shorter transcripts (up to position ∼440, i.e., ending immediately 3′ of SL1) formed tight dimers through the SL1 apical loop (Dirac et al., 2001; Lanchy et al., 2003a). A possible explanation relies on the fact that HIV-2 RNA secondary structure forms a LRI between a C-rich region upstream of the PBS and a G-rich region overlapping the *gag* translation initiation codon termed C box-G box interaction (CGI), homologous to the HIV-1 U5:AUG interaction (compare **Figures 7B, 8B**). Interestingly, while this latter was proposed to promote HIV-1 RNA dimerization through SL1 (Dirac et al., 2001; Lanchy et al., 2003a), the CGI seems to prevent formation of tight HIV-2 RNA dimers since targeting the CGI with oligonucleotides restores SL1-dependent dimerization *in vitro* (Lanchy et al., 2003a,b). Moreover, incubation of RNA fragments either at high temperature (>50°C) or in presence of HIV-2 NC has been shown to destabilize the CGI and restore SL1-dependent tight dimerization *in vitro* (Lanchy and Lodmell, 2002; Lanchy et al., 2003b). Interestingly, in addition to the apical loop of SL1, an upstream 10 nt-long conserved palindromic sequence (termed PAL) also contributes to *in vitro* RNA tight dimer formation (**Figure 8C**). Indeed, by using antisense oligonucleotides targeting the SL1 apical DIS or PAL, this latter was shown to serve as an alternative stabilization element when the apical DIS is repressed (Lanchy et al., 2003a). Importantly, *in vitro* SHAPE and biochemical analyses revealed that stem B of SL1 (**Figure 8C**), formed by base-pairing between the 3′-end of PAL and a region downstream of SL1 stem A (**Figure 8C**), regulates HIV-2 RNA dimerization (Baig et al., 2007; Purzycka et al., 2011). Indeed, in the proposed model, upon opening of SL1 stem B through the chaperone activity of Gag/NC, the PAL region becomes available to build interstrand contacts. Interestingly, a comparative study revealed that the dimerization properties of HIV-1, HIV-2 and SIV RNAs are regulated through the stability of SL1 stem B (Baig et al., 2008).

Consistently with these *in vitro* results, mutations in the PAL region strongly impaired HIV-2 RNA dimerization and infectivity *in cellula*, while mutations in the SL1 apical loop had limited impact (L’Hernault et al., 2007, 2012). Similarly, the integrity of SL1 stem B was shown to be crucial for HIV-2 gRNA packaging (Lanchy and Lodmell, 2007). Finally, it was also proposed that, *in cellula*, translation of HIV-2 gRNA by the ribosome may disrupt the CGI to allow RNA dimerization, a notion consistent with a study proposing that HIV-2 genome is packaged co-translationally (Griffin et al., 2001). However, this *cis*-packaging model was recently challenged and it was elegantly shown by visualization HIV-2 RNA in individual particles that *trans*-packaging is the major mechanism of HIV-2 RNA packaging, as it was observed for HIV-1 (Ni et al., 2011).

## Spumaviruses Genome Dimerization

Spumaretroviruses, or foamy viruses (FVs), have the largest genome in the *Retroviridae* family (∼12 kb) and are singularly different from other retroviruses. Amongst these differences, FV Gag proteins do not present the NC Cys-His zinc fingers, a hallmark of other retroviral Gag proteins, but have instead Gly-Arg rich domains binding nucleic acids with equal affinity and required for viral replication (Yu et al., 1996; Linial, 1999). Furthermore, reverse transcription is a late event in FV replication cycle and infectious viral particles contain DNA genomes (Linial, 1999; Yu et al., 1999). Despite these differences, three different sites (SI, SII, and SIII) were shown to contribute to *in vitro* dimerization of RNA fragments corresponding to the 5′ region of FV gRNA (Erlwein et al., 1997). Amongst these domains, SII maps to positions 391–410 and contains a highly conserved 10-nts UCCUAGGA palindrome that is crucial for *in vitro* RNA dimerization since antisense oligonucleotides or mutations targeting this motif abolish RNA dimer formation (Erlwein et al., 1997; Cain et al., 2001). SI and SIII also contribute to RNA dimerization, but to a lesser extent since antisense oligonucleotide targeting of these elements alone failed to completely abrogate *in vitro* RNA dimerization of primate FV 5′-RNA fragments (Erlwein et al., 1997; Cain et al., 2001). However, targeting bovine FV (BFV) SI element by antisense oligonucleotides inhibited *in vitro* RNA dimerization similarly to what was observed when targeting the SII palindrome (Yu et al., 2007).

Importantly, mutation of the SII palindrome in cell culture resulted in a strong viral replication defect, even though genome packaging was not affected (Park and Mergia, 2000; Cain et al., 2001), differently from other retroviruses such as HIV-1 which showed defects in gRNA packaging when mutation of the DIS occurred (Berkhout and van Wamel, 1996; Paillart et al., 1996a; Laughrea et al., 1997, 1999; Houzet et al., 2007). Since it is well known that RNA dimerization plays an important role in reverse transcription (Paillart et al., 1996a; Berkhout et al., 1998; Parent et al., 2000), it seems likely that this replication defect may come from reverse transcription defects (Park and Mergia, 2000).

## *In Cellula* Genome Dimerization: Where and When?

Retroviruses select specifically two copies of their genome from the pool of cellular and viral spliced RNAs for packaging. Since aberrant gRNA dimerization may interfere with different steps in the retroviral life cycle, including viral assembly, the timing and localization of this process must be finely regulated within the host cell. An important question has been whether two monomers are packaged and dimerize during/after viral assembly, or if a pre-formed RNA dimer is selected and packaged into viral particles. However, since gRNA dimerization and packaging are interconnected events (Aagaard et al., 2004; Houzet et al., 2007; Moore et al., 2007; Miyazaki et al., 2010a; Mailler et al., 2016), gRNA dimerization was supposed to initiate upon gRNA recognition by the Gag precursor. In the following section, we will review the current understanding of the spatio-temporal regulation of retroviral RNA dimerization.

Various studies using protease-deficient (PR^-^) virions or different Gag mutants suggested that HIV-1 gRNA is packaged as two monomers (Shehu-Xhilaga et al., 2001; Song et al., 2007). It is, however, possible that gRNA extraction protocols may disrupt immature loose dimers, especially when the stability of RNA mutants is reduced (Höglund et al., 1997). Although it has been reported that, in some cell types, HIV-1 gRNA DIS mutants presenting packaging defects can still be dimeric (Laughrea et al., 1997; Hill et al., 2003), a majority of studies showed that disruption of SL1 kissing-loop interface impaired or affected gRNA packaging (Berkhout and van Wamel, 1996; Paillart et al., 1996a; Houzet et al., 2007; Moore et al., 2007). Moreover, duplication of HIV-1 DLS leads to production of partially monomeric genomes, due to gRNA circularization by self-dimerization (Sakuragi et al., 2001, 2007), contradicting the notion that HIV-1 packages its genome as RNA monomers. The comparison of HIV-1 gRNA structure *in cellula* and *in viro* revealed little structural rearrangements between these two conditions (Paillart et al., 2004), and recombination analyses supported the notion that gRNA dimerization initiates in the cytoplasm (Moore et al., 2009).

An extensive set of analyses support the idea that gRNA dimerization occurs prior budding of viral particle (Moore et al., 2007; Jouvenet et al., 2009; Moore and Hu, 2009; Sardo et al., 2015; Ferrer et al., 2016). Because retroviral assembly involves only two copies of gRNA, detecting RNA dimerization in cells is challenging. Several bio-imaging approaches based on fluorescence have been used to address this issue. To allow specific gRNA observation *in cellula*, the viral genome is labeled by incorporating numerous stem-loops that specifically bind the coat protein of MS2 bacteriophage (Bertrand et al., 1998; Fusco et al., 2003), or the phage lambda protein λN22 (Daigle and Ellenberg, 2007) fused to fluorescent protein(s). Although it was suggested that HIV-1 gRNA migrates at the plasma membrane as a pre-formed dimer (Jouvenet et al., 2009; Chen et al., 2016), further *ex vivo* analyses showed that HIV-1 gRNA dimer reaches the plasma membrane in association with low-order multimers of Gag precursor (Jouvenet et al., 2008, 2009; Kutluay and Bieniasz, 2010; Kutluay et al., 2014). Importantly, Ferrer et al. (2016) recently combined Fluorescence *In Situ* Hybridization (FISH), TIRF-M, 3D-super-resolution microscopy and Fluorescence Cross-Correlation Spectroscopy (FCCS) to show that HIV-1 gRNA dimerization already occurs in the cytosol but that RNA dimers are more easily detected at the plasma membrane. One possible explanation could be the concentration of Gag at the plasma membrane, which might promote gRNA dimer stabilization through the chaperone activity of its NC domain (Ferrer et al., 2016). In this context, Hu and co-workers used a similar approach and proposed that HIV-1 gRNA dimerization would occur preferentially at the plasma membrane (Chen et al., 2016). Their comparison between TIRF-M and *in silico* simulations showed that RNA heterodimerization would occur at frequencies similar to those observed in Ferrer et al. (2016) study (∼10–15%). Since the co-localization frequencies increased at the plasma membrane in a Gag-dependent manner, they thus proposed that HIV-1 genome diffuses into the cytoplasm and reaches the plasma membrane as a monomer. However, a common point for both studies resides in the differences between heterodimers frequencies at the plasma membrane (∼10–15% co-localization) and in virions (∼40–50%) (Chen et al., 2009; Ferrer et al., 2016), suggestive of an RNA dimer stabilization step at the plasma membrane *prior* to encapsidation. It is, however, possible that technical difficulties for the detection of RNA molecules in a highly dynamic environment, such as the cytosol and the plasma membrane, compared to the stable environment within viral particles, could affect these estimations. Further analyses are definitively needed to clarify spatiotemporal HIV-1 gRNA dimerization.

Finally, studies suggest that MuLV gRNA dimerization, unlike HIV-1, occurs in the nucleus. Indeed, heterodimerization is enhanced when two proviruses are spatially close, suggesting that MuLV gRNA dimerization is coupled to transcription and splicing processes (Maurel and Mougel, 2010). Consistent with these observations, MuLV RNAs transcribed from the same locus form dimers at high frequencies (Flynn et al., 2004; Kharytonchyk et al., 2005; Rasmussen and Pedersen, 2006; Maurel et al., 2007). Interestingly, mutations of RNA elements involved in dimerization or packaging processes impact the intracellular transport of viral genome and result in aberrant accumulation in the nucleus or in the cytoplasm (Basyuk et al., 2005; Smagulova et al., 2005).

## RNA Dimerization and Reverse Transcription/Recombination

Reverse transcription is initiated at the PBS immediately 3′ to the 5′ copy of the R (repeat) region of the genome by the viral RT enzyme and generates the complete viral cDNA with duplicated LTR. In order to achieve cDNA synthesis, the negative strong-stop DNA must be translocated from the 5′ to the 3′-end of the gRNA during the first strand transfer occurring in an intra- or inter-molecular manner (Panganiban and Fiore, 1988; Hu and Temin, 1990; Berkhout and van Wamel, 1996). During reverse transcription, recombination occurs as the RT enzyme switches between the two RNA templates. This process is due to RNA sequence homology and promoted by particular RNA structures, nicks in the viral genome or RT pausing (Galetto and Negroni, 2005). Importantly, recombination provides a mechanism for genome repair or, in the case of a heterodiploid genome, a way to increase genetic diversity by genomes shuffling. This leads to immune escape and drug resistance, especially in the case of HIV-1 (Morris et al., 1999; Rambaut et al., 2004), for which it has previously been shown that gRNA heterodimerization rate can reach ∼40–50% *in viro* (Chen et al., 2009; Ferrer et al., 2016), and that recombination occurs at least three times during a single viral replication cycle (Zhuang et al., 2002; Schlub et al., 2010, 2014; Smyth et al., 2014).

Genomic RNA dimerization may play an important role during reverse transcription since RSV mutant particles containing monomeric genomes display a 100-fold decrease in cDNA synthesis (Parent et al., 2000) and heat-induced dissociation of HIV-1 gRNA dimers inhibits the first strand transfer (Berkhout et al., 1998). In MuLV, template switching occurs preferentially at direct RNA–RNA interactions mediated by palindromic sequences (Mikkelsen et al., 1998a,b, 2000). In the case of a co-infection by two genetically different viruses, the subsequent packaging of a heterozygous dimer increases the viral diversity by enabling the production of new recombinant strains (Mikkelsen et al., 2000). In HIV-1, *trans*-complementary DIS mutants showed that the recombination rate is enhanced when two palindromic mutants can form Watson–Crick base-pairs (from 50 to 90% depending on the mutants) (Moore et al., 2009). Similarly, both the *in vitro* and *in cellula* HIV-1 recombination rates are directly linked to the dimeric state of the viral genome (Balakrishnan et al., 2001, 2003; Sakuragi et al., 2015, 2016). Taken together, these findings highlight the central role of gRNA dimerization in the reverse transcription and recombination processes.

## RNA Dimerization as a Potential Antiretroviral Target

While the current antiretroviral treatments have proved successful in extending the life expectancy of infected patients, the increasing number of multi-drug resistant HIV-1 mutants highlights the need for the discovery of new antiretroviral drugs. In this context, gRNA dimerization is an attractive target since it is crucial for several key steps in the retrovirus life cycle. Interestingly, the crystal structure of the HIV-1 kissing-loop complex revealed strong structural similarities with the 16S ribosomal A site, a natural aminoglycoside binding site (Ennifar et al., 2003). Indeed, some aminoglycosides, such as neomycin and lividomycin, bind the HIV-1 kissing-loop complex *in vitro* with high affinity and strongly stabilize it, preventing its conversion to an extended (Ennifar et al., 2003, 2006, 2007; Bernacchi et al., 2007). Interestingly, footprint analysis also revealed that the DIS of HIV-1 gRNA is also protected by aminoglycosides in cells and in virions (Ennifar et al., 2006, 2007), thus providing an interesting basis for the development of new antiretroviral strategies aiming at targeting retroviral gRNA dimerization (Ennifar et al., 2007, 2013; Blond et al., 2014).

## Concluding Remarks

Retroviral gRNA dimerization is a highly conserved mechanism in retroviruses, and plays a critical role in several key steps of their replicative cycle. RNA dimerization, which is a pre-requisite for packaging, plays a critical role in the generation of multi-drug resistant recombinant viruses during reverse transcription. In this context, a general feature of retroviruses is the existence of complex structural switches regulating the transition between a loose kissing-loop complex to a stable extended duplex conformation, and the equilibrium between gRNA translation and dimerization for packaging. Nevertheless, *in vitro* structural models most likely do not exactly reflect the *in vivo* situation and, despite recent progresses, much remains to be unraveled to reach complete understanding of the RNA dimerization process. This understanding could also allow the development of new antiretroviral strategies that are essential to counterbalance the increasing prevalence of multi-drug resistant viruses.

## Author Contributions

ND, RM, J-CP, and SB conceived the review topic. ND drafted the manuscript and generated the figures. SB, J-CP, and RM corrected and edited the manuscript. All authors read and approved the final version of the manuscript.

## Conflict of Interest Statement

The handling Editor declared a shared affiliation, though no other collaboration, with all authors.
